# Edge Mostar Indices of Cacti Graph With Fixed Cycles

**DOI:** 10.3389/fchem.2021.693885

**Published:** 2021-07-09

**Authors:** Farhana Yasmeen, Shehnaz Akhter, Kashif Ali, Syed Tahir Raza Rizvi

**Affiliations:** ^1^ Department of Mathematics, COMSATS University Islamabad, Lahore, Pakistan; ^2^ School of Natural Science, National University of Science and Technology, Islamabad, Pakistan

**Keywords:** topological invariants, Mostar invariant, edge Mostar invariant, cacti graphs, graph theory

## Abstract

Topological invariants are the significant invariants that are used to study the physicochemical and thermodynamic characteristics of chemical compounds. Recently, a new bond additive invariant named the Mostar invariant has been introduced. For any connected graph ℋ, the edge Mostar invariant is described as $Mo_{e}\left(ℋ\right)=\sum_{gx\in E\left(ℋ\right)}\left|m_{ℋ}\left(g\right)-m_{ℋ}\left(x\right)\right|$, where $m_{ℋ}\left(g\right)\left(\text{or }m_{ℋ}\left(x\right)\right)$ is the number of edges of ℋ lying closer to vertex *g* (or *x*) than to vertex *x* (or *g*). A graph having at most one common vertex between any two cycles is called a cactus graph. In this study, we compute the greatest edge Mostar invariant for cacti graphs with a fixed number of cycles and *n* vertices. Moreover, we calculate the sharp upper bound of the edge Mostar invariant for cacti graphs in ℭ(n,s), where *s* is the number of cycles.

## 1 Introduction

Let ℋ=(V(ℋ),E(ℋ)) be a simple, undirected, and connected graph with the vertex set V(ℋ) and the edge set E(ℋ). The degree of g∈V(ℋ), represented as $deg_{ℋ}\left(g\right)$, is described as the number of edges directly linked with *g*. The neighbors of a vertex *g* in ℋ is the set of all of its adjacent vertices in ℋ. For g,x∈V(ℋ), the number of edges in the shortest path between two vertices *g* and *x* is called the distance between them and is expressed as $d_{ℋ}\left(g,x\right)$. A pendent vertex *p* in ℋ is a vertex with degree one, and an edge having one pendent vertex as one of its end vertices is called a pendent edge. The set of all pendent vertices of ℋ is represented as $P_{ℋ}$, and the set of all pendent vertices adjacent to a fix vertex *g* is represented as $P_{ℋ}\left(g\right)$. An edge in ℋ is presented as a cut edge if, by deleting that edge, the graph is converted into exactly two components. Let $P_{n}$, $C_{n}$, and $S_{n}$ be used for the representation of the path, the cycle, and the star with order *n*.

In the fields of chemical sciences, mathematical chemistry, chemical graph theory, and pharmaceutical science, topological invariants are of significant importance because of their definitional use. The physicochemical properties of chemical structures can be forecasted by using topological invariants. A numerical value related to biological activity, chemical reactivity, and physical properties of chemical structures is known as a topological invariant. Topological invariants are mainly separated into different manners like degree, distance, eccentricity, and spectrum. A distance-based invariant is a topological invariant based on the distance between the vertices or edges of a given graph. The Wiener invariant (Wiener, 1947) is the most significant oldest topological invariant that belongs to distance-based invariants, and the Harary invariant (Mihalić and Trinajstić, 1992) and the Balaban invariant (Zhou and Trinajstić, 2008) also belong to distance-based invariants. Degree-based invariants are another well-studied group of invariants. The first degree-based invariant was introduced as the Randić invariant (Randic, 1975). A rich theory of distance- and degree-based invariants is mentioned in (Li and Shi, 2008; Gutman, 2013; Knor et al., 2014; Knor et al., 2015). The recently introduced Mostar invariant (Došlić et al., 2018) belongs to bound additive invariants as they capture the relevant properties of a graph by summing up the contributions of individual edges (Vukičević and Gašperov, 2010; Vukičević, 2011). Peripherality is one such property that could be of interest. An edge is a peripheral edge if there are many more vertices closer to one of its end vertices than to the other one. In short, for an edge gx in ℋ, the greatest value of absolute difference of the cardinality of vertices closer to *g* than to *x*, presented by $n_{ℋ}\left(g\right)$, and the cardinality of vertices closer to *x* than to *g*, denoted by $n_{ℋ}\left(x\right)$, indicates a peripheral position of gx in ℋ. The Mostar invariant of a graph ℋ is defined as follows:

$$Mo_{v}\left(ℋ\right)=\underset{e=gx\in E\left(ℋ\right)}{\sum }\left|n_{ℋ}\left(g\right)-n_{ℋ}\left(x\right)\right|,$$ (1)

and this represents a global measure of peripherality of a graph ℋ. Došlić et al. (2018) determined the Mostar invariant of the benzenoid system. Tratnik proved that the Mostar invariant of the weighted graph can be deduced in the form of the Mostar invariant of quotient graphs (Tratnik, 2019). Arockiaraj et al. (2019) introduced the edge Mostar invariant as follows:

$$Mo_{e}\left(ℋ\right)=\underset{e=gx\in E\left(ℋ\right)}{\sum }\left|m_{ℋ}\left(g\right)-m_{ℋ}\left(x\right)\right|,$$ (2)

where $m_{ℋ}\left(g\right)\left(\text{or }m_{ℋ}\left(x\right)\right)$ is the cardinality of edges closer to *g* (or *x*) than to *x* (or *g*).Akhter et al. (2021) computed the Mostar indices for the molecular graphs of SiO_2_ layer structures and the melem chain with the help of the cut method. Liu et al. (2020) found the extremal values of the edge Mostar invariant of cacti graphs. Imran et al. (2020) found the edge Mostar invariant of chemical structures and nanostructures using graph operations. Arockiaraj et al. (2020) calculated the weighted Mostar indices of molecular peripheral shapes with applications in graphene, graphyne, and graphdiyne nanoribbons. Liu et al. (2020) determined the maximum edge Mostar index of cacti graphs with the following given conditions.

**Theorem 1.1.** *Let* G∈ℭ(n,s) *be a connected graph:*

• *if* n≥10 *and* n<4s *, then* $Mo_{e}\left(G\right)\leq 2n^{2}-8n+\left(24-4n\right)s$ *with equality if and only if* $G≅G^{n}\left(\underset{4s-n}{\underset{︸}{3,3,3,\ldots 3}},\underset{n-3s}{\underset{︸}{4,4,4\ldots 4}}\right)$,
• *if* n≥10 *and* n≥4s *, then* $Mo_{e}\left(G\right)\leq n^{2}-n-12s$ *with equality if and only if* $G≅G^{n}\left(4,4,\ldots 4\right)$,
• *if* n=9 *, then* $Mo_{e}\left(G\right)7=72-12s$ *with equality if and only if* $G≅G_{9}$, *and*
• *if* n<9 *, then* $Mo_{e}\left(G\right)\leq n^{2}-n-\left(n+3\right)s$ *with equality if and only if* $G≅G^{n}\left(3,3,3,\ldots 3\right)$.

Liu et al. (2020) determined the second maximum edge Mostar index of cacti graphs with the following given conditions.

**Theorem 1.2.** *Let* $G\in ℭ\left(n,s\right)\backslash {ℭ}_{0}\left(n,s\right)$ *with* n≥10 *and* n≥4s>0 *:*

• $Mo_{e}\left(G\right)\leq 89-12s$ *for* n=10 *with equality if and only if* $G≅G\left(3,\underset{s-1}{\underset{︸}{4,4,4\ldots 4}}\right)$ *,*
• $Mo_{e}\left(G\right)\leq 108-12s$ *for* n=11 *with equality if and only if* $G≅G\left(3,\underset{s-1}{\underset{︸}{4,4,4\ldots 4}}\right)$ *, and*
• $Mo_{e}\left(G\right)\leq n^{2}-n-12s-2$ *with equality if and only if* $G≅G_{1}\left(n,s\right)$ *.*

For more results related to Mostar and edge Mostar invariants, see (Hayat and Zhou, 2019a; Akhter, 2019; Tepeh, 2019; Akhter et al., 2020; Dehgardi and Azari, 2020; Deng and Li, 2020; Ghorbani et al., 2020; Huang et al., 2020; Deng and Li, 2021a; Deng and Li, 2021b).

 A connected graph is a cactus if all its blocks are either edges or cycles, that is, any two of its cycles have at most one common vertex. Until now, many results in chemistry and graph theory related to the cacti have been acquired. The first three smallest Gutman invariants among the cacti have been determined by Chen (2016). Using the Zagreb invariants, Li et al. (2012) found the upper and lower bounds of the cacti. The bounds of the Harary invariant related to cacti have been found by Wang and Kang (2013). The extremal cacti having the greatest hyper-Wiener invariant have been characterized by Wang and Tan (2015). The extremal graphs with the greatest and smallest vertex PI invariants among all cacti with a fixed number of vertices have been determined by Wang et al. (2016). The sharp upper bound of the Mostar invariant for cacti of order *n* with *s* cycles has been given by Hayat and Zhou (2019b), and they also found the greatest Mostar invariant for all *n*-vertex cacti. For more results related to cacti graphs, see (Liu et al., 2016; Wang and Wei, 2016; Wang, 2017).

 Motivated by the results of chemical invariants and their applications, it may be interesting to characterize the cacti with the greatest and smallest edge Mostar invariants for some fixed parameters. In this study, we consider the cacti with a fixed number of cycles and find the greatest edge Mostar invariant for all the *n*-vertex cacti. In the end, we give a sharp upper bound of the edge Mostar invariant for these cacti.

## 2 Main Results

**FIGURE 1:**
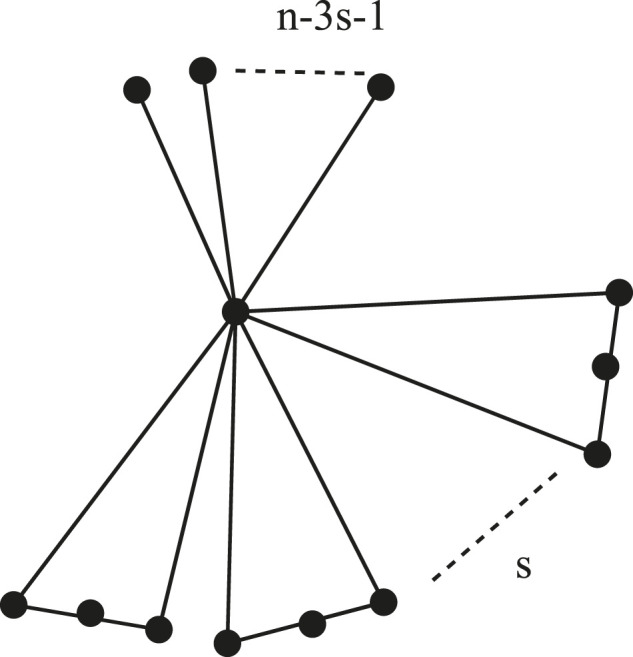
Graph $\hat{ℭ}\left(n,s\right)$, for n≥3s+2, s≥2 and for n≥9, s=1.

Let ℭ(n) be the set of all cacti graphs of order n≥2 and ℭ(n,s) be the set of all cacti graphs of order n≥2 with the number of cycles *s*. Let $\hat{ℭ}\left(n,s\right)\in ℭ\left(n,s\right)$ be the *n*-vertex cactus, for n≥3s+2, s≥2 and for n≥9, s=1, consisting of *s* number of $C_{4}$ and n−3s−1 pendent edges such that every $c_{4}$ and pendent edge has exactly one vertex in common (see Figure 1).

In this section, we derive the greatest value of cacti graphs for the edge Mostar invariant. First of all, some basic lemmas are proved so that the main result can be proved easily.

Proposition 2.1. (Imran et al., 2020) *The edge Mostar invariant of a path* $P_{n}$ *and a cycle* $C_{n}$ *with n vertices is* $Mo_{e}\left(P_{n}\right)=\left[\frac{{\left(n-1\right)}^{2}}{2}\right]$ *and* $Mo_{e}\left(C_{n}\right)=0$ *, respectively.*

 In Lemma 2.1, we establish a graph $G_{2}$ by converting a cut edge uv into a pendent edge uw in $G_{1}$, such that the new graph $G_{2}$ has a greater edge Mostar invariant.

**FIGURE 2:**
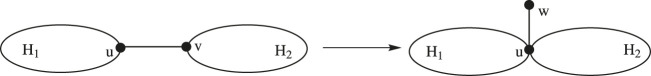
Graphs $G_{1}$ and $G_{2}$ of Lemma 2.1.

Lemma 2.1: *Consider two connected graphs* $H_{1}$ *and* $H_{2}$ *such that they are connected to each other by an edge* uv *, where* $u\in V\left(H_{1}\right)$ *and* $v\in V\left(H_{2}\right)$ *, and acquired the graph* $G_{1}$ *. Now, we construct the graph* $G_{2}$ *by deleting the cut edge* uv *and attaching a pendent edge* uw *at vertex u in* $G_{1}$ (*see* Figure 2). *Then* $Mo_{e}\left(G_{1}\right)<Mo_{e}\left(G_{2}\right)$ *.*

Proof : Let $H_{1}$ and $H_{2}$ be the subgraphs of $G_{1}$, as shown in Figure 2. By the construction of $G_{2}$, the number of closer edges of the end vertices of a fixed edge of $H_{1}$ and $H_{2}$ in $G_{1}$ remains the same in $G_{2}$, respectively. Therefore, for an edge $gx\in E\left(H_{l}\right)$, where l∈{1,2}, we have the following:

$$m_{G_{1}}\left(g\right)=m_{G_{2}}\left(g\right),\;\;m_{G_{1}}\left(x\right)=m_{G_{2}}\left(x\right)$$ (3)

For the cut edge uv in $G_{1}$ and the pendent edge uw in $G_{2}$, we have the following:

$$m_{G_{1}}\left(u\right)=\left|E\left(H_{1}\right)\right|,\;m_{G_{1}}\left(v\right)=\left|E\left(H_{2}\right)\right|,$$

$$m_{G_{2}}\left(u\right)=\left|E\left(H_{1}\right)\right|+\left|E\left(H_{2}\right)\right|,\;m_{G_{2}}\left(w\right)=0.$$ (4)

Using the definition of the edge Mostar invariant and substituting the values from Eqs 3, 4 , we acquire the following:

$$\begin{array}{c} Mo_{e}\left(G_{1}\right)-Mo_{e}\left(G_{2}\right)=\left|m_{G_{1}}\left(u\right)-m_{G_{1}}\left(v\right)\right|+\overset{2}{\underset{l=1}{\sum }}\underset{gx\in E\left(H_{l}\right)}{\sum }\left|m_{G_{1}}\left(g\right)-m_{G_{1}}\left(x\right)\right|-\left|m_{G_{2}}\left(u\right)-m_{G_{2}}\left(w\right)\right|\\ -\overset{2}{\underset{l=1}{\sum }}\underset{gx\in E\left(H_{l}\right)}{\sum }\left|m_{G_{2}}\left(g\right)-m_{G_{2}}\left(x\right)\right|\\ =\left|\left|E\left(H_{1}\right)\right|+\left|E\left(H_{2}\right)\right|\right|+\overset{2}{\underset{l=1}{\sum }}\underset{gx\in E\left(H_{l}\right)}{\sum }\left|m_{G_{1}}\left(g\right)-m_{G_{1}}\left(x\right)\right|-\left|\left|E\left(H_{2}\right)\right|-\left|E\left(H_{1}\right)\right|\right|\\ -\overset{2}{\underset{l=1}{\sum }}\underset{gx\in E\left(H_{l}\right)}{\sum }\left|m_{G_{1}}\left(g\right)-m_{G_{1}}\left(x\right)\right|\\ =\left\|E\left(H_{1}\right)|-|E\left(H_{2}\right)\right\|-\left\|E\left(H_{1}\right)|+|E\left(H_{2}\right)\right\|. \end{array}$$

There are two cases:

1. if $\left|E\left(H_{1}\right)\right|>\left|E\left(H_{2}\right)\right|$, then we get $\left|E\left(H_{1}\right)\right|-\left|E\left(H_{2}\right)\right|-\left|E\left(H_{1}\right)\right|-\left|E\left(H_{2}\right)\right|=-2\left|E\left(H_{2}\right)\right|<0,\text{ and}$
2. if $\left|E\left(H_{1}\right)\right|<\left|E\left(H_{2}\right)\right|$, then we get $-\left|E\left(H_{1}\right)\right|+\left|E\left(H_{2}\right)\right|-\left|E\left(H_{1}\right)\right|-\left|E\left(H_{2}\right)\right|=-2\left|E\left(H_{1}\right)\right|<0$.

In either case, we acquire $Mo_{e}\left(G_{1}\right)-Mo_{e}\left(G_{2}\right)<0$.

This completes the proof. ∎

Next, we establish a new $G_{2}$ graph from $G_{1}$ by moving all pendent edges, all $C_{4}$ cycles, and all $C_{3}$ cycles from different vertices of a fixed cycle $C_{s}$ to a unique vertex, such that the new graph has a larger edge Mostar invariant.

**FIGURE 3:**
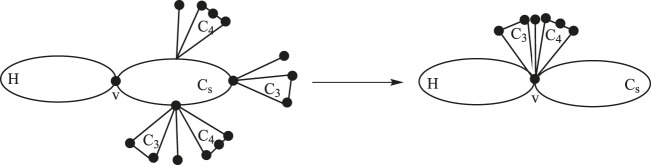
Graphs $G_{1}$ and $G_{2}$ of Lemma 2.2.

Lemma 2.2: *Let* G *be a cyclic graph constructed by attaching* $r_{i}$ *, for* $r_{i}\geq 0$ *, number of pendent vertices,* $t_{i}$ *, for* $t_{i}\geq 0$ *, number of* $C_{4}$ *cycles and* $m_{i}$ *, for* $m_{i}\geq 0$ *, number of* $C_{3}$ *cycles, at the vertices* $v_{i}$ *, for* 1≤i≤s−1 *, of* $C_{s}$ *, where* s≥3 *. Consider a graph H having a common vertex* v∈V(H) *with* G *and present it by* $G_{1}$ *. We construct* $G_{2}$ *from* $G_{1}$ *by removing all the pendent vertices,* $C_{4}$ *’s, and* $C_{3}$ *’s of* G *and attaching them at v (see* Figure 3 *). Then, we have* $Mo_{e}\left(G_{1}\right)<Mo_{e}\left(G_{2}\right)$ *.*

Proof: Suppose that the vertices of $C_{s}$ are $v_{0}\left(=v\right),v_{1},v_{2},\ldots ,v_{s-1}$ and there are $r_{i}$ number of pendent edges, $t_{i}$ number of $C_{4}$ cycles, and $m_{i}$ number of $C_{3}$ cycles rooted at $v_{i}$, for 1≤i≤s−1, in $G_{1}$. By the construction of $G_{2}$, the number of closer edges of the end vertices of a fixed edge of *H* in $G_{1}$ remains the same in $G_{2}$. Therefore, for any edge $u_{1}u_{2}\in E\left(H\right)$, we have the following:

$$m_{G_{1}}\left(u_{1}\right)=m_{G_{2}}\left(u_{1}\right),\;\;m_{G_{1}}\left(u_{2}\right)=m_{G_{2}}\left(u_{2}\right).$$ (5)

For the pendent edges $v_{i}u$ rooted on $v_{i}$, for 1≤i≤s−1 and u∈P(G), in $G_{1}$, we have the following:

$$m_{G_{1}}\left(v_{i}\right)=\left|E\left(H\right)\right|+\left|E\left(G\right)\right|-1,m_{G_{1}}\left(u\right)=0=m_{G_{2}}\left(u\right)m_{G_{2}}\left(v=v_{0}\right)=\left|E\left(H\right)\right|+\left|E\left(G\right)\right|-1.$$ (6)

For every $C_{4}$ cycle rooted on a fixed vertex $v_{i}$, for 1≤i≤s−1, the edge set is $\left\{w_{0}w_{1},w_{1}w_{2},w_{2}w_{3},w_{3}w_{0}\right\}$, and then, there are the following cases:

1. For $w_{i}w_{i+1}$, i=0,1, we have $m_{G_{1}}\left(w_{i}\right)=\left|E\left(H\right)\right|+\left|E\left(G\right)\right|-3=m_{G_{2}}\left(w_{i}\right)$ and $m_{G_{1}}\left(w_{i+1}\right)=1=m_{G_{2}}\left(w_{i+1}\right)$.
2. For $w_{2}w_{3}$, we have $m_{G_{1}}\left(w_{3}\right)=\left|E\left(H\right)\right|+\left|E\left(G\right)\right|-3=m_{G_{2}}\left(w_{3}\right)$ and $m_{G_{1}}\left(w_{2}\right)=1=m_{G_{2}}\left(w_{2}\right)$.
3. For $w_{0}w_{3}$, we have $m_{G_{1}}\left(w_{0}\right)=\left|E\left(H\right)\right|+\left|E\left(G\right)\right|-3=m_{G_{2}}\left(w_{0}\right)$ and $m_{G_{1}}\left(w_{3}\right)=1=m_{G_{2}}\left(w_{3}\right)$.

For every $C_{3}$ cycle rooted on a fixed vertex $v_{i}$, for 1≤i≤s−1, the edge set is $\left\{g_{0}g_{1},g_{1}g_{2},g_{2}g_{3},g_{3}g_{0}\right\}$, and then, there are the following cases:

1. For $g_{0}g_{1}$, we have $m_{G_{1}}\left(g_{0}\right)=\left|E\left(H\right)\right|+\left|E\left(G\right)\right|-2=m_{G_{2}}\left(g_{0}\right)$ and $m_{G_{1}}\left(g_{1}\right)=1=m_{G_{2}}\left(g_{1}\right)$.
2. For $g_{1}g_{2}$, we have $m_{G_{1}}\left(g_{3}\right)=m_{G_{1}}\left(g_{2}\right)$ and $m_{G_{2}}\left(g_{3}\right)=m_{G_{2}}\left(g_{2}\right)$.
3. For $g_{0}g_{2}$, we have $m_{G_{1}}\left(g_{0}\right)=\left|E\left(H\right)\right|+\left|E\left(G\right)\right|-2=m_{G_{2}}\left(g_{0}\right)$ and $m_{G_{1}}\left(g_{2}\right)=1=m_{G_{2}}\left(g_{2}\right)$.

Suppose $C_{s}$ is an even cycle; then there are the following cases:

1. For $v_{0}v_{1}$, we have $m_{G_{1}}\left(v_{0}\right)=\left|E\left(H\right)\right|+\frac{s}{2}-1+\sum_{p=\frac{s}{2}+1}^{s-1}\left(r_{p}+t_{p}+m_{p}\right)$ and $m_{G_{1}}\left(v_{1}\right)=\frac{s}{2}-1+\sum_{p=1}^{\frac{s}{2}}\left(r_{p}+t_{p}+m_{p}\right)$.
2. For $v_{i}v_{i+1}$, where $1\leq i\leq \frac{s}{2}-1$, we have $m_{G_{1}}\left(v_{i}\right)=\left|E\left(H\right)\right|+\frac{s}{2}-1+\sum_{p=\frac{s}{2}+i+1}^{s-1}\left(r_{p}+t_{p}+m_{p}\right)+\sum_{p=1}^{i}\left(r_{p}+t_{p}+m_{p}\right)$ and $m_{G_{1}}\left(v_{i+1}\right)=\frac{s}{2}-1+\sum_{p=i+1}^{\frac{s}{2}+i}\left(r_{p}+t_{p}+m_{p}\right).$
3. For $v_{i}v_{i+1}$, where $\frac{s}{2}\leq i\leq s-2$, we have $m_{G_{1}}\left(v_{i}\right)=\frac{s}{2}-1+\sum_{p=i-\left(\frac{s}{2}-1\right)}^{i}\left(r_{p}+t_{p}+m_{p}\right)$ and $m_{G_{1}}\left(v_{i+1}\right)=\left|E\left(H\right)\right|+\frac{s}{2}-1+\sum_{p=i+1}^{s-1}\left(r_{p}+t_{p}+m_{p}\right)+\sum_{p=1}^{i-\frac{s}{2}}\left(r_{p}+t_{p}+m_{p}\right)$.
4. For $v_{0}v_{s-1}$, we have $m_{G_{1}}\left(v_{0}\right)=\left|E\left(H\right)\right|+\frac{s}{2}-1+\sum_{p=1}^{\frac{s}{2}-1}\left(r_{p}+t_{p}+m_{p}\right)$ and $m_{G_{1}}\left(v_{s-1}\right)=\frac{s}{2}-1+\sum_{p=\frac{s}{2}}^{s-1}\left(r_{p}+t_{p}+m_{p}\right)$.
5. For $v_{i}v_{i+1}$, where $0\leq i\leq \frac{s}{2}-1$, we have $m_{G_{2}}\left(v_{i}\right)=|E\left(H\right)|+\frac{s}{2}-1+\sum_{p=1}^{s-1}\left(r_{p}+t_{p}+m_{p}\right)$ and $m_{G_{2}}\left(v_{i+1}\right)=\frac{s}{2}-1$.
6. For $v_{i}v_{i+1}$, where $\frac{s}{2}\leq i\leq s-2$, we have $m_{G_{2}}\left(v_{i}\right)=\frac{s}{2}-1$ and $m_{G_{2}}\left(v_{i+1}\right)=\left|E\left(H\right)\right|+\frac{s}{2}-1+\sum_{p=1}^{s-1}\left(r_{p}+t_{p}+m_{p}\right)$.
7. For $v_{0}v_{s-1}$, we have $m_{G_{2}}\left(v_{0}\right)=\left|E\left(H\right)\right|+\frac{s}{2}-1+\sum_{p=1}^{s-1}\left(r_{p}+t_{p}+m_{p}\right)$ and $m_{G_{2}}\left(v_{s-1}\right)=\frac{s}{2}-1$.

Substituting the values from Eqs 5, 6 and the information from all the cases above in the definition of the edge Mostar invariant, we acquire the following:

$$\begin{array}{l} Mo_{e}\left(G_{1}\right)-Mo_{e}\left(G_{2}\right)=\underset{u_{1}u_{2}\in E\left(H\right)}{\sum }\left|m_{G_{1}}\left(u_{1}\right)-m_{G_{1}}\left(u_{2}\right)\right|+\underset{v_{i}u\in E\left(G\right),u\in P_{G}\left(v_{i}\right)}{\sum }\left|m_{G_{1}}\left(v_{i}\right)-m_{G_{1}}\left(u\right)\right|\\ +\sum_{i=0}^{3}\sum_{w_{i}w_{i+1}\in E\left(C_{4}\right)}\left|m_{G_{1}}\left(w_{i}\right)-m_{G_{1}}\left(w_{i+1}\right)\right|+\left|m_{G_{1}}\left(w_{0}\right)-m_{G_{1}}\left(w_{3}\right)\right|\\ +\sum_{i=0}^{2}\sum_{g_{i}g_{i+1}\in E\left(C_{3}\right)}\left|m_{G_{1}}\left(g_{i}\right)-m_{G_{1}}\left(g_{i+1}\right)\right|+\left|m_{G_{1}}\left(g_{0}\right)-m_{G_{1}}\left(g_{2}\right)\right|+\sum_{i=0}^{s-2}|m_{G_{1}}\left(v_{i}\right)\\ -m_{G_{1}}\left(v_{i+1}\right)|+\left|m_{G_{1}}\left(v_{0}\right)-m_{G_{1}}\left(v_{s-1}\right)\right|-\sum_{u_{1}u_{2}\in E\left(H\right)}\left|m_{G_{2}}\left(u_{1}\right)-m_{G_{2}}\left(u_{2}\right)\right|\\ -\sum_{vu\in E\left(G\right),u\in P_{G}\left(v_{i}\right)}\left|m_{G_{2}}\left(u\right)-m_{G_{2}}\left(v\right)\right|-\sum_{i=0}^{3}\sum_{w_{i}w_{i+1}\in E\left(C_{4}\right)}\left|m_{G_{2}}\left(w_{i}\right)-m_{G_{2}}\left(w_{i+1}\right)\right|\\ -\left|m_{G_{2}}\left(w_{0}\right)-m_{G_{2}}\left(w_{3}\right)\right|-\sum_{i=0}^{2}\sum_{g_{i}g_{i+1}\in E\left(C_{3}\right)}\left|m_{G_{2}}\left(g_{i}\right)-m_{G_{2}}\left(g_{i+1}\right)\right|-|m_{G_{2}}\left(g_{0}\right)\\ -m_{G_{2}}\left(g_{2}\right)|-\sum_{i=0}^{s-2}\left|m_{G_{2}}\left(v_{i}\right)-m_{G_{2}}\left(v_{i+1}\right)\right|-\left|m_{G_{2}}\left(v_{0}\right)-m_{G_{2}}\left(v_{s-1}\right)\right|\\ =\sum_{u_{1}u_{2}\in E\left(H\right)}\left|m_{{ℋ}_{1}}\left(u_{1}\right)-m_{{ℋ}_{1}}\left(u_{2}\right)\right|+r\left|\left|E\left(H\right)\right|+\left|E\left(G\right)\right|-1\right|+4t|\left|E\left(H\right)\right|\\ +\left|E\left(G\right)\right|-4|+2m\left|\left|E\left(H\right)\right|+\left|E\left(G\right)\right|-3\right|+|\left|E\left(H\right)\right|+\frac{s}{2}-1\\ +\sum_{p=\frac{s}{2}+i+1}^{s-1}\left(r_{p}+t_{p}+m_{p}\right)+\frac{s}{2}-1-\frac{s}{2}+1-\sum_{p=i+1}^{\frac{s}{2}+i}\left(r_{p}+t_{p}+m_{p}\right)|+\sum_{i=\frac{s}{2}}^{s-2}|\sum_{p=i-\left(\frac{s}{2}-1\right)}^{i}\left(r_{p}+t_{p}+m_{p}\right)\\ +\frac{s}{2}-1-\frac{s}{2}+1-\sum_{p=i+1}^{s-1}\left(r_{p}+t_{p}+m_{p}\right)-\left|E\left(H\right)\right|-\sum_{p=1}^{i}\left(r_{p}+t_{p}+m_{p}\right)|+|\left|E\left(H\right)\right|+\frac{s}{2}-1. \end{array}\begin{array}{l} +\sum_{p=\frac{s}{2}+1}^{s-1}\left(r_{p}+t_{p}+m_{p}\right)-\frac{s}{2}+1-\sum_{p=1}^{\frac{s}{2}}\left(r_{p}+t_{p}+m_{p}\right)|-\sum_{u_{1}u_{2}\in E\left(H\right)}\left|m_{{ℋ}_{1}}\left(u_{1}\right)-m_{{ℋ}_{1}}\left(u_{2}\right)\right|-r|\left|E\left(H\right)\right|\\ +\left|E\left(G\right)\right|-1|-4t|\left|E\left(H\right)\right|+\left|E\left(G\right)\right|-4|-2m\left|\left|E\left(H\right)\right|+\left|E\left(G\right)\right|-3\right|-\sum_{i=0}^{\frac{s}{2}-1}|\left|E\left(H\right)\right|+\frac{s}{2}-1+\overset{s-1}{\underset{p=1}{\sum }}\\ \left(r_{p}+t_{p}+m_{p}\right)-\frac{s}{2}+1|-\sum_{i=\frac{s}{2}}^{s-2}\left|+\frac{s}{2}-1-\left|E\left(H\right)\right|-\frac{s}{2}+1-\sum_{p=1}^{s-1}\left(r_{p}+t_{p}+m_{p}\right)\right|-|\left|E\left(H\right)\right|+\frac{s}{2}-1\\ +\sum_{p=1}^{s-1}\left(r_{p}+t_{p}+m_{p}\right)-\frac{s}{2}+1|\\ \leq \left|E\left(H\right)\right|+\sum_{p=1}^{s-1}\left(r_{p}+t_{p}+m_{p}\right)+\sum_{i=1}^{\frac{s}{2}-1}\left(\left|E\left(H\right)\right|+\sum_{p=1}^{s-1}\left(r_{p}+t_{p}+m_{p}\right)\right)+\sum_{i=\frac{s}{2}}^{s-2}\left(\left|E\left(H\right)\right|+\sum_{p=1}^{s-1}\left(r_{p}+t_{p}+m_{p}\right)\right)\\ +\left|E\left(H\right)\right|+\sum_{p=1}^{s-1}\left(r_{p}+t_{p}+m_{p}\right)-\sum_{i=0}^{\frac{s}{2}-1}\left(\left|E\left(H\right)\right|+\sum_{p=1}^{s-1}\left(r_{p}+t_{p}+m_{p}\right)\right)-\sum_{i=\frac{s}{2}}^{s-2}\left(\left|E\left(H\right)\right|+\overset{s-1}{\underset{p=1}{\sum }}\left(r_{p}+t_{p}+m_{p}\right)\right)\\ -\left(\left|E\left(H\right)\right|+\sum_{p=1}^{s-1}\left(r_{p}+t_{p}+m_{p}\right)\right)\\ \leq \left|E\left(H\right)\right|+r+t+m+\sum_{i=1}^{s-2}(|E\left(H\right)|+r+t+m)-\sum_{i=0}^{s-2}(|E\left(H\right)|+r+t+m)\\ \leq \left|E\left(H\right)\right|+r+t+m+\left(s-2\right)(|E\left(H\right)|+r+t+m)-\left(s-1\right)\left(\left|E\left(H\right)\right|+r+t+m\right)\\ \leq 0. \end{array}$$

The proof for an odd cycle $C_{s}$ is similar to that above; therefore, we omit it here.

This completes the proof. ∎

In Lemma 2.3, we establish a new graph $G_{2}$ from a given graph $G_{1}$ by replacing $C_{q}$ with $C_{4}$ and attaching q−4 pendent edges in $G_{1}$ such that the new graph has a greater edge Mostar invariant.

**FIGURE 4:**
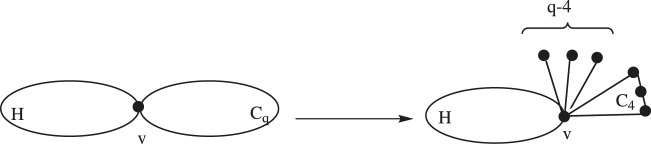
Graphs $G_{1}$ and $G_{2}$ of Lemma 2.3.

Lemma 2.3: *Consider a graph H having a common vertex* v∈V(H) *with* $C_{q}$ *such that* ${\text{deg}}_{H}\left(v\right)\geq 3$ *and* q≥5 *, and denote it as* $G_{1}$ *. Let* $G_{2}$ *be the graph acquired from* $G_{1}$ *by replacing* $C_{q}$ *with* $C_{4}$ *and attaching* q−4 *pendent edges at* v∈V(H) *(see* Figure 4 *). Then, we have* $Mo_{e}\left(G_{1}\right)\leq Mo_{e}\left(G_{2}\right)$ *.*

Proof: Let *H* be a subgraph of $G_{1}$ and the vertices of $C_{q}$ be $v_{0}\left(=v\right),v_{1},v_{2},\ldots ,v_{q-1}$, as shown in Figure 4. By the construction of $G_{2}$, the number of closer edges of the end vertices of a fixed edge of *H* in $G_{1}$ remains the same in $G_{2}$. Therefore, for any edge $u_{1}u_{2}\in E\left(H\right)$, we have the following:

$$m_{G_{1}}\left(u_{1}\right)=m_{G_{2}}\left(u_{1}\right),\;m_{G_{1}}\left(u_{2}\right)=m_{G_{2}}\left(u_{2}\right).$$ (7)

Suppose *q* is even; then there are three cases:

1. For $v_{i}v_{i+1}$, where $0\leq i\leq \frac{q}{2}-1$, we have $m_{G_{1}}\left(v_{i}\right)=\left|E\left(H\right)\right|+\frac{q}{2}-1$ and $m_{G_{1}}\left(v_{i+1}\right)=\frac{q}{2}-1$.
2. For $v_{i}v_{i+1}$, where $\frac{q}{2}\leq i\leq q-2$, we have $m_{G_{1}}\left(v_{i}\right)=\frac{q}{2}-1$ and $m_{G_{1}}\left(v_{i+1}\right)=\left|E\left(H\right)\right|+\frac{q}{2}-1$.
3. For $v_{0}v_{q-1}$, we have $m_{G_{1}}\left(v_{0}\right)=\left|E\left(H\right)\right|+\frac{q}{2}-1$ and $m_{G_{1}}\left(v_{q-1}\right)=\frac{q}{2}-1$.

Suppose *q* is odd; then there are three cases:

1. For $v_{i}v_{i+1}$, where $0\leq i\leq \frac{q}{2}-1$, we have $m_{G_{1}}\left(v_{i}\right)=\left|E\left(H\right)\right|+\frac{q-1}{2}$ and $m_{G_{1}}\left(v_{i+1}\right)=\frac{q-1}{2}$.
2. For $v_{i}v_{i+1}$, where $\frac{q}{2}\leq i\leq q-2$, we have $m_{G_{1}}\left(v_{i}\right)=\frac{q-1}{2}$ and $m_{G_{1}}\left(v_{i+1}\right)=\left|E\left(H\right)\right|+\frac{q-1}{2}$.
3. For $v_{0}v_{q-1}$, we have $m_{G_{1}}\left(v_{0}\right)=\left|E\left(H\right)\right|+\frac{q-1}{2}$ and $m_{G_{1}}\left(v_{q-1}\right)=\frac{q-1}{2}.$

In $G_{2}$, for any pendent edge $vv_{i}$, where 4≤i≤q−1, rooted at *v*, we have the following:

$$m_{G_{2}}\left(v\right)=|E\left(H\right)|+q-1,\;\;m_{G_{2}}\left(v_{i}\right)=0.$$ (8)

For $v_{0}v_{1},v_{1}v_{2},v_{2}v_{3},v_{3}v_{0}$ in $G_{2}$, there are the following cases:

1. For $v_{i}v_{i+1}$, i=0,1, we have $m_{G_{2}}\left(v_{i}\right)=\left|E\left(H\right)\right|+q-3$ and $m_{G_{2}}\left(v_{i+1}\right)=1$.
2. For $v_{2}v_{3}$, we have $m_{G_{2}}\left(v_{3}\right)=\left|E\left(H\right)\right|+q-3$ and $m_{G_{2}}\left(v_{2}\right)=1$.
3. For $v_{0}v_{3}$, we have $m_{G_{2}}\left(v_{0}\right)=|E\left(H\right)|+q-3$ and $m_{G_{2}}\left(v_{3}\right)=1$.

Case 1: When *q* is even, using the definition of the edge Mostar invariant and substituting the values from Eqs 7, 8 and the cases above, we get the following:

$$\begin{array}{l} Mo_{e}\left(G_{1}\right)-Mo_{e}\left(G_{2}\right)=\sum_{u_{1}u_{2}\in E\left(H\right)}\left|m_{G_{1}}\left(u_{1}\right)-m_{G_{1}}\left(u_{2}\right)\right|+\sum_{i=0}^{q-2}\left|m_{G_{1}}\left(v_{i}\right)-m_{G_{1}}\left(v_{i+1}\right)\right|+|m_{G_{1}}\left(v_{0}\right)\\ -m_{G_{1}}(v_{q-1})|-\sum_{u_{1}u_{2}\in E\left(H\right)}\left|m_{G_{2}}\left(u_{1}\right)-m_{G_{2}}\left(u_{2}\right)\right|-\sum_{i=4}^{q-1}\left|m_{G_{2}}\left(v\right)-m_{G_{2}}\left(v_{i}\right)\right|\\ -\left|m_{G_{2}}\left(v_{0}\right)-m_{G_{2}}\left(v_{1}\right)\right|-\left|m_{G_{2}}\left(v_{1}\right)-m_{G_{2}}\left(v_{2}\right)\right|-\left|m_{G_{2}}\left(v_{2}\right)-m_{G_{2}}\left(v_{3}\right)\right|-\left|m_{G_{2}}\left(v_{3}\right)-m_{G_{2}}\left(v_{0}\right)\right|\\ =\sum_{u_{1}u_{2}\in E\left(H\right)}\left|m_{G_{1}}\left(u_{1}\right)-m_{G_{1}}\left(u_{2}\right)\right|+\sum_{i=0}^{\frac{q}{2}-1}\left|\left|E\left(H\right)\right|+\frac{q}{2}-1-\left(\frac{q}{2}-1\right)\right|\\ +\sum_{i=\frac{q}{2}}^{q-2}\left|\frac{q}{2}-1-(|E\left(H\right)|+\frac{q}{2}-1)\right|+\left|\left|E\left(H\right)\right|+\frac{q}{2}-1-\left(\frac{q}{2}-1\right)\right|\\ -\sum_{u_{1}u_{2}\in E\left(H\right)}|m_{G_{2}}\left(u_{1}\right)-m_{G_{2}}\left(u_{2}\right)|-\sum_{i=4}^{q-1}\left|\left|E\left(H\right)\right|+q-1-0)\right|-4\left|\left|E\left(H\right)\right|+q-3-1\right|\\ =\sum_{u_{1}u_{2}\in E\left(H\right)}\left|m_{G_{1}}\left(u_{1}\right)-m_{G_{1}}\left(u_{2}\right)\right|+\sum_{i=0}^{\frac{q}{2}-1}\left|E\left(H\right)\right|+\sum_{i=\frac{q}{2}}^{q-2}\left|E\left(H\right)\right|+\left|E\left(H\right)\right|\\ -\sum_{u_{1}u_{2}\in E\left(H\right)}\left|m_{G_{1}}\left(u_{1}\right)-m_{G_{1}}\left(u_{2}\right)\right|-\sum_{i=4}^{q-1}\left|\left|E\left(H\right)\right|+q-1\right|-4\left|\left|E\left(H\right)\right|+q-4\right|\leq q\left|E\left(H\right)\right|-\left(q-4\right)\left|E\left(H\right)\right|-\left(q-4\right)q+\left(q-4\right)-4\left|E\left(H\right)\right|-4q+16\leq -q^{2}+q+12<0. \end{array}$$

Case 2: When *q* is odd, using the definition of the edge Mostar invariant and substituting the values from Eqs 7, 8 and the cases above, we get the following:

$$\begin{array}{c} Mo_{e}\left(G_{1}\right)-Mo_{e}\left(G_{2}\right)=\sum_{u_{1}u_{2}\in E\left(H\right)}\left|m_{G_{1}}\left(u_{1}\right)-m_{G_{1}}\left(u_{2}\right)\right|+\sum_{i=0}^{q-2}\left|m_{G_{1}}\left(v_{i}\right)-m_{G_{1}}\left(v_{i+1}\right)\right|+|m_{G_{1}}\left(v_{0}\right)\\ -m_{G_{1}}(v_{v_{q-1}})|-\sum_{u_{1}u_{2}\in E\left(H\right)}\left|m_{G_{2}}\left(u_{1}\right)-m_{G_{2}}\left(u_{2}\right)\right|-\sum_{i=4}^{q-1}\left|m_{G_{2}}\left(v\right)-m_{G_{2}}\left(v_{i}\right)\right|-\left|m_{G_{2}}\left(v_{0}\right)-m_{G_{2}}\left(v_{1}\right)\right|-\left|m_{G_{2}}\left(v_{1}\right)-m_{G_{2}}\left(v_{2}\right)\right|-\left|m_{G_{2}}\left(v_{2}\right)-m_{G_{2}}\left(v_{3}\right)\right|\\ -\left|m_{G_{2}}\left(v_{3}\right)-m_{G_{2}}\left(v_{0}\right)\right|, \end{array}$$

$$\begin{array}{l} Mo_{e}\left(G_{1}\right)-Mo_{e}\left(G_{2}\right)=\sum_{u_{1}u_{2}\in E\left(H\right)}\left|m_{G_{1}}\left(u_{1}\right)-m_{G_{1}}\left(u_{2}\right)\right|+\sum_{i=0}^{\frac{q}{2}-1}\left|\left|E\left(H\right)\right|+\frac{q-1}{2}-\left(\frac{q-1}{2}\right)\right|\\ +\sum_{i=\frac{q}{2}}^{q-2}\left|\frac{q-1}{2}-(|E\left(H\right)|+\frac{q-1}{2})\right|+\left|\left|E\left(H\right)\right|+\frac{q-1}{2}-\left(\frac{q-1}{2}\right)\right|\\ -\sum_{u_{1}u_{2}\in E\left(H\right)}\left|m_{G_{2}}\left(u_{1}\right)-m_{G_{2}}\left(u_{2}\right)\right|-\sum_{i=4}^{q-1}\left|\left|E\left(H\right)\right|+q-1-0)\right|-4\left|\left|E\left(H\right)\right|+q-3-1\right|\\ =\sum_{u_{1}u_{2}\in E\left(H\right)}\left|m_{G_{1}}\left(u_{1}\right)-m_{G_{1}}\left(u_{2}\right)\right|+\sum_{i=0}^{\frac{q}{2}-1}\left|E\left(H\right)\right|+\sum_{i=\frac{q}{2}}^{q-2}\left|E\left(H\right)\right|+\left|E\left(H\right)\right|\\ -\sum_{u_{1}u_{2}\in E\left(H\right)}\left|m_{G_{1}}\left(u_{1}\right)-m_{G_{1}}\left(u_{2}\right)\right|-\sum_{i=4}^{q-1}\left|\left|E\left(H\right)\right|+q-1\right|-4\left|\left|E\left(H\right)\right|+q-4\right|\leq q\left|E\left(H\right)\right|-\left(q-4\right)\left|E\left(H\right)\right|-\left(q-4\right)q+\left(q-4\right)-4\left|E\left(H\right)\right|-4q+16\leq -q^{2}+q+12<0. \end{array}$$

This completes the proof. ∎

**FIGURE 5:**
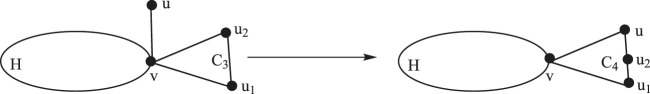
Graphs $G_{1}$. and $G_{2}$ of Lemma 2.4.

Lemma 2.4: *Consider a graph H having a common vertex* v∈V(H) *with* $C_{3}$ *and at least one pendent edge* vu *, and this graph is presented as* $G_{1}$ *. Let* $G_{2}$ *be the graph obtained from* $G_{1}$ *by replacing* $C_{3}$ *and* vu *with* $C_{4}$ *(see* Figure 5 *). Then, we have* $Mo_{e}\left(G_{1}\right)<Mo_{e}\left(G_{2}\right)$ *.*

Proof: By the construction of $G_{2}$, the number of closer edges of the end vertices of a fixed edge of *H* in $G_{1}$ remains the same in $G_{2}$. Therefore, for any edge $u_{1}u_{2}\in E\left(H\right)$, we have the following:

$$m_{G_{1}}\left(u_{1}\right)=m_{G_{2}}\left(u_{1}\right),\;\;m_{G_{1}}\left(u_{2}\right)=m_{G_{2}}\left(u_{2}\right).$$ (9)

There are the following cases in $G_{1}$:

1. For pendent edge $uv\in E\left(G_{1}\right)$, we have $m_{G_{1}}\left(v\right)=\left|E\left(H\right)\right|+3$ and $m_{G_{1}}\left(u\right)=0$.
2. For $vu_{1}\in E\left(C_{3}\right)$, we have $m_{G_{1}}\left(v\right)=\left|E\left(H\right)\right|+2$ and $m_{G_{1}}\left(u_{1}\right)=1$.
3. For $vu_{2}\in E\left(C_{3}\right)$, we have $m_{G_{1}}\left(v\right)=\left|E\left(H\right)\right|+2$ and $m_{G_{1}}\left(u_{2}\right)=1$.
4. For $u_{1}u_{2}\in E\left(C_{3}\right)$, we have $m_{G_{1}}\left(u_{1}\right)=m_{G_{1}}\left(u_{2}\right)$.

By the construction of $G_{2}$, we have the following:

1. For $uv\in E\left(C_{4}\right)$, we have $m_{G_{2}}\left(u\right)=1$ and $m_{G_{1}}\left(v\right)=\left|E\left(H\right)\right|+1$.
2. For $vu_{1}\in E\left(C_{4}\right)$, we have $m_{G_{2}}\left(v\right)=\left|E\left(H\right)\right|+1$ and $m_{G_{2}}\left(u_{1}\right)=1$.
3. For $u_{1}u_{2}\in E\left(C_{4}\right)$, we have $m_{G_{2}}\left(u_{1}\right)=\left|E\left(H\right)\right|+1$ and $m_{G_{2}}\left(u_{2}\right)=1$.
4. For $u_{2}u\in E\left(C_{4}\right)$, we have $m_{G_{2}}\left(u_{2}\right)=1$ and $m_{G_{2}}\left(u\right)=\left|E\left(H\right)\right|+1$.

Using the definition of the edge Mostar invariant and substituting the values from cases, we get the following:

$$\begin{array}{c} Mo_{e}\left(G_{1}\right)-Mo_{e}\left(G_{2}\right)=\sum_{u_{1}u_{2}\in E\left(H\right)}\left|m_{G_{1}}\left(u_{1}\right)-m_{G_{1}}\left(u_{2}\right)\right|+\sum_{i=0}^{q-2}\left|m_{G_{1}}\left(v_{i}\right)-m_{G_{1}}\left(v_{i+1}\right)\right|+|m_{G_{1}}\left(v_{0}\right)\\ -m_{G_{1}}\left(v_{q-1}\right)|-\sum_{u_{1}u_{2}\in E\left(H\right)}\left|m_{G_{2}}\left(u_{1}\right)-m_{G_{2}}\left(u_{2}\right)\right|-\sum_{i=4}^{q-1}\left|m_{G_{2}}\left(v\right)-m_{G_{2}}\left(v_{i}\right)\right|\\ -\left|m_{G_{2}}\left(v_{0}\right)-m_{G_{2}}\left(v_{1}\right)\right|-\left|m_{G_{2}}\left(v_{1}\right)-m_{G_{2}}\left(v_{2}\right)\right|-\left|m_{G_{2}}\left(v_{2}\right)-m_{G_{2}}\left(v_{3}\right)\right|\\ -\left|m_{G_{2}}\left(v_{3}\right)-m_{G_{2}}\left(v_{0}\right)\right|, \end{array}$$

$$\begin{array}{c} Mo_{e}\left(G_{1}\right)-Mo_{e}\left(G_{2}\right)=\sum_{u_{1}u_{2}\in E\left(H\right)}\left|m_{G_{1}}\left(u_{1}\right)-m_{G_{1}}\left(u_{2}\right)\right|+\sum_{i=0}^{\frac{q}{2}-1}\left|\left|E\left(H\right)\right|+\frac{q-1}{2}-\left(\frac{q-1}{2}\right)\right|\\ +\sum_{i=\frac{q}{2}}^{q-2}\left|\frac{q-1}{2}-\left(\left|E\left(H\right)\right|+\frac{q-1}{2}\right)\right|+\left|\left|E\left(H\right)\right|+\frac{q-1}{2}-\left(\frac{q-1}{2}\right)\right|\\ -\sum_{u_{1}u_{2}\in E\left(H\right)}\left|m_{G_{2}}\left(u_{1}\right)-m_{G_{2}}\left(u_{2}\right)\right|-\sum_{i=4}^{q-1}\left|\left|E\left(H\right)\right|+q-1-0)\right|-4\left|\left|E\left(H\right)\right|+q-3-1\right|\\ =\sum_{u_{1}u_{2}\in E\left(H\right)}\left|m_{G_{1}}\left(u_{1}\right)-m_{G_{1}}\left(u_{2}\right)\right|+\sum_{i=0}^{\frac{q}{2}-1}\left|E\left(H\right)\right|+\sum_{i=\frac{q}{2}}^{q-2}\left|E\left(H\right)\right|+\left|E\left(H\right)\right|\\ -\sum_{u_{1}u_{2}\in E\left(H\right)}\left|m_{G_{1}}\left(u_{1}\right)-m_{G_{1}}\left(u_{2}\right)\right|-\sum_{i=4}^{q-1}\left|\left|E\left(H\right)\right|+q-1\right|-4\left|\left|E\left(H\right)\right|+q-4\right|\\ \leq q\left|E\left(H\right)\right|-\left(q-4\right)\left|E\left(H\right)\right|-\left(q-4\right)q+\left(q-4\right)-4\left|E\left(H\right)\right|-4q+16\\ \leq -q^{2}+q+12<0. \end{array}$$

This completes the proof. ∎

Theorem 2.1: *Among all the cacti graphs in* ℭ(n,s) *, the cactus* $\hat{ℭ}\left(n,s\right)$ *, for* n≥3s+2 *,* s≥2 *and for* n≥9 *,* s=1 *, shown in* Figure 1 *has the largest edge Mostar invariant. Thus, for any cactus* G∈ℭ(n,s) *, we have* $Mo_{e}\left(G\right)\leq Mo_{e}\left(\tilde{ℭ}\left(n,s\right)\right)$ *.*

Proof: Let G∈ℭ(n,s) be a cactus graph where s≥0 and n≥2. If $G≇\tilde{ℭ}\left(n,s\right)$ and G has a cut edge, then repeatedly applying Lemma 2.1, we get a sequence of new cacti graphs $G_{1},G_{2},\ldots ,G_{b}$, where $G_{b}$ is a cactus without any cut edge, such that $Mo_{e}\left(G\right)<Mo_{e}\left(G_{1}\right)<Mo_{e}\left(G_{2}\right)<\cdots <Mo_{e}\left(G_{b}\right)$. Now, if $G_{b}≇\tilde{ℭ}\left(n,s\right)$ and $G_{b}$ have a cyclic subgraph G' that is constructed by attaching $r_{i}$, for $r_{i}\geq 0$, number of pendent vertices, $t_{i}$, for $t_{i}\geq 0$, number of $C_{4}$ cycles and $m_{i}$, for $m_{i}\geq 0$, number of $C_{3}$ cycles, at the vertices $v_{i}$, for 1≤i≤s−1, of $C_{s}$, where s≥3, then by applying Lemma 2.2 repeatedly, we acquire a sequence of cacti graphs $G_{b},G_{b_{1}},G_{b_{2}},\ldots ,G_{b_{k}}$ satisfying $Mo_{e}\left(G_{b}\right)<Mo_{e}\left(G_{b_{1}}\right)<Mo_{e}\left(G_{b_{2}}\right)<\cdots <Mo_{e}\left(G_{b_{k}}\right)$, where $G_{b_{k}}$ is a cactus graph such that every vertex of cycles of $G_{b_{k}}$ has degree 2 except common vertices. If $G_{b_{k}}≇\tilde{ℭ}\left(n,s\right)$ and $G_{b_{k}}$ have a cycle $C_{q}$, for q≥5, then by applying Lemma 2.3 repeatedly, we acquire a sequence of cacti graphs $G_{b_{k}},G_{b_{k_{1}}},G_{b_{k_{2}}},\ldots ,G_{b_{k_{c}}}$ satisfying $Mo_{e}\left(G_{b_{k}}\right)<Mo_{e}\left(G_{b_{k_{1}}}\right)<Mo_{e}\left(G_{b_{k_{2}}}\right)<\ldots <Mo_{e}\left(G_{b_{k_{c}}}\right)$, where $G_{b_{k_{c}}}≅\tilde{ℭ}\left(n,s\right)$. If $G_{b_{k_{c}}}$ has a triangle $C_{3}$ and at least one pendent edge vw, then by using Lemma 2.4, we construct a cactus graph $G_{b_{k_{c}}}^{\prime }$ with a cycle $C_{4}$ and get the greatest Mostar invariant and then $Mo_{e}\left(G_{b_{k_{c}}}\right)$.

 This completes the proof. ∎

 By Theorem 2.1 and simple calculation, we have the following results:

Corollary 2.1. *Let* G∈ℭ(n,s) *be a cactus graph with* n≥2 *and number of cycles s; then we have the following:*

$$Mo_{e}\left(G\right)\leq \{\begin{array}{cc} n^{2}-3n+2, & if\;s=0\;and\;n\geq 2,\\ n^{2}-n-12, & if\;s=1\;and\;n\geq 9,\\ n^{2}+\left(2s-3\right)n+s^{2}-15s+2, & if\;s\geq 2\;and\;n\geq 3s+2, \end{array}$$

equality holds if $G≅\tilde{ℭ}\left(n,s\right)$.

## 3 Conclusion

The ongoing direction of numerical coding of the fundamental chemical structures with topological descriptors has been substantiated as completely victorious. This approach substantiates the contrast, quarry, renewal, interpretation, and swift troupe of chemical structures within enormous particularities. Eventually, topological descriptors can lead to productive measures for quantitative structure–activity relationships (QSARs) and quantitative structure–property relationships (QSPRs), which are imitations that identify chemical structures with chemical reactivity, physical properties, or biological activity. The edge Mostar index is a newly proposed quantity; it has not been used in physicochemical or biological research. Recently, a work (Imran et al., 2020) has been completed in this direction for chemical structures and nanostructures using graph operations. The authors have found the edge Mostar indices of nanostructures. Motivated by these results, we have studied the maximum edge Mostar invariant of the *n*-vertex cacti graphs with a fixed number of cycles in this study. For this, we have proved some lemmas in which we use the transformation of graphs and some calculations. In future, we want to find the largest and smallest edge Mostar invariants of the *n*-vertex cacti graphs with some fixed parameters other than the number of cycles.

## Data Availability

The original contributions presented in the study are included in the article/Supplementary Material; further inquiries can be directed to the corresponding author.
